# Predictive factors for efficacy and safety in refractive surgery for myopia

**DOI:** 10.1371/journal.pone.0208608

**Published:** 2018-12-14

**Authors:** Nir Gomel, Shay Negari, Joseph Frucht-Pery, Denise Wajnsztajn, Eyal Strassman, Abraham Solomon

**Affiliations:** 1 Department of Ophthalmology, Hadassah-Hebrew University Medical Center, Jerusalem, Israel; 2 Department of Public Health, Hadassah-Hebrew University Medical Center, Jerusalem, Israel; Universidade do Minho, PORTUGAL

## Abstract

**Purpose:**

To evaluate the predictive factors for safety and efficacy in laser refractive surgery for myopia

**Setting:**

A singular refractive surgery center, at a University-affiliated tertiary medical center.

**Design:**

Retrospective cohort study

**Methods:**

Study population—A total 8,775 eyes having laser refractive laser procedures for myopia (in4,623 patients).

Observation procedures–Using a prospective database of refractive procedures performed over the span of 13 years, variables such as gender, age, type of surgery, date of surgery, pre-operative corneal thickness and Spherical Equivalent (SEQ) were evaluated.

Main outcome measures—Proportion of patients with Safety index higher than 0.85 and Efficacy index higher than 0.80.

**Results:**

91.9% and 86.0% of all evaluated eyes were above the safety and efficacy cut-off levels, respectively. Younger age was significantly correlated with safety and efficacy indices above the cut-off levels (p<0.001). Male gender was significantly correlated with efficacy above the cut-off level (p<0.001). Myopic eyes with lower SEQ were associated with both safety (p = 0.002) and efficacy (p<0.001) indices above the cut-offs. The surgical procedure was found to significantly affect the outcome only using univariate analysis: Safety was higher in Photorefractive Keratectomy (PRK), while Efficacy was higher in Laser Assisted In Situ Keratomileusis (LASIK) (p<0.001, respectively) but no difference was found using multivariate analysis. Safety index above the cut-off level increased over the years (p<0.001).

**Conclusions:**

Efficacy in refractive surgery for myopia is correlated with younger age, male gender and low myopia. Safety is correlated with younger age, low myopia and increases over the years. Multivariate analysis found no differences between PRK and LASIK regarding safety and efficacy.

## Introduction

Refractive surgery is being performed for more than 30 years and is considered to be the safest and most favorable option for vision correction in myopic eyes [1].

These procedures are known to be very safe and highly effective, yet there are still many concerns regarding their safety and efficacy [2–3]. Loss of Best Corrected Visual Acuity (BSCVA) of more than one line was reported in 0.06%-0.1% and up to 0.4% in some reports. Severe complications were reported with very low rate–ectasia in up to 0.6%, stromal flap complications in up to 1.0% (in LASIK (laser assisted in situ keratomileusis) procedures) and recurrent corneal erosions in up to 0.08%. Postoperative UCVA (uncorrected Visual acuity) of 20/20 or better was reported in 70% of procedures and UCVA of 20/40 or better was reported in 90–95% of procedures [3–7].

Although laser refractive procedures have been practiced worldwide for almost 30 years, no study investigating the predictive factors for success in both safety and efficacy has been published yet. Success rates of these procedures, differences between PRK (Photo Refractive Keratectomy) and LASIK procedures, and post-operative complications and their treatments are commonly reported. Many studies investigated the outcome of refractive procedures, and some of these included large cohorts such as Baviera J et al. and several Cochrane reviews [4,8–12]. However, none of them has investigated the preoperative factors predicting the safety and efficacy outcomes. Therefore, in our study, we evaluated the predictive factors for safety and efficacy of refractive surgery procedures.

The purpose of this study was to evaluate the predictive factors for safety and efficacy in laser refractive surgery for myopia. We investigated these factors using a large database of patients at the refractive center of the Department of Ophthalmology in Hadassah-Hebrew University Medical Center. The Hadassah Refractive center is characterized by a small fixed team of surgeons and using the same manufacturer for the excimer laser hardware and software over the years. The laser system in use was Bausch and Lomb Keracor 217z excimer laser and since 2007 the Zyoptix wavefront guided system was used. These characteristics enable reliable analysis of retrospective data with relatively minimal bias.

This study included a relatively large cohort of patients which were operated over a span of 13 years. A novel analysis system was used to evaluate safety and efficacy, using clinically significant cut-off values for the safety and efficacy indices.

## Methods

This cohort study adhered to the tenets of the Declaration of Helsinki and was approved by the Hadassah Medical Center IRB (no. 0083-17-HMO). All data were fully anonymized before we accessed them.

### Data collection

Data was collected from the medical records at the Hadassah Refractive Surgery Center. A retrospective database of 22,610 laser refractive procedures for myopia (in 11,834 patients) which were performed at Hadassah Refractive Surgery Center during the years 2002 through 2015 was available for this study. Data from pre-operative and follow-up visits was collected and analyzed. For each eye, parameters such as gender, age, type of surgery, date of surgery, pre-operative central corneal thickness and pre-operative Spherical Equivalent (SEQ) were evaluated. UCVA and BCVA were collected from follow-up visits.

Exclusion criteria included: patients who had refractive surgeries for hyperopia. Patients with incomplete data were excluded from the analysis. Manual scanning was performed to identify and delete duplications and mistakes. Missing data was completed by using the patients’ files. Only surgeries with follow-up visits between three to twelve months were included in this study and among those visits, the latest follow-up visit was used. Patients with keratoconus or other corneal pathologies were not operated.

### Study population

The initial sample included data from 22,610 consecutive laser refractive procedures for myopia and hyperopia. Excluded procedures included 6,883 due to insufficient data details (BCVA, UCVA, surgery type), 828 hyperopic procedures, and 5,837 with insufficient follow up of 3–12 months. Following these exclusions, the sample included data on 8,775 refractive procedures for myopia.

The pre-operative SEQ was divided into four groups based on the magnitude of myopia: low myopia (0 to -3 D), moderate myopia (-3.01 to -6 D), high myopia (-6.01 to -9 D) and very high myopia (>-9.01 D) [2,3,9,13]. Treatment dates were divided into three time periods based on the introduction of Wavefront technology: 2002 to 2006 when Wavefront technology was not available, 2007–2010 when Wavefront technology was optional, and 2011–2015 when it was mandatory in all patients.

The decision whether to perform LASIK or PRK surgery was made according to the usual criteria including the expected residual stromal bed thickness, the corneal topography, and the patients occupation and lifestyle. We wanted to preserve a residual stromal bed thickness of 250 μ at the end of the surgery. Therefore, patients with high myopia or with thin corneas underwent PRK surgery most of the times. Among patients with asymmetric topography at the inferior-superior axis, we avoided performing LASIK in order to prevent ectasia. Moreover, if a patient preferred one of the procedures due to his/her occupation or lifestyle, we accepted their requests.

### Outcome measures

The two major outcome measures were the Safety Index and Efficacy Index at last follow-up visit. The Safety Index was defined as BSCVA after treatment divided by BSCVA before treatment (BSCVA post/BSCVA pre). The Efficacy Index was defined as UCVA after treatment divided by BSCVA before treatment (UCVA post/BCVA pre).

Cut-off levels of 0.85 for the Safety index and 0.80 for the Efficacy index were set to determine successful results at last follow-up for these two indices, respectively. Values with safety index above the 0.85 cut-off indicate loss of less than two lines, while values below the 0.85 cut-off level indicate loss of two or more lines, which was not acceptable by most refractive surgeons for BSCVA after surgery. While a loss of one line may seem acceptable, loss of two lines usually indicates a lack of success regarding the safety of these procedures [4,14–15].

Values with efficacy index above the 0.8 cut-off indicate loss of up to two lines of UCVA, which most patients could be tolerable, while values below the 0.8 cut-off level indicate loss of more than two lines, where both surgeons and patients may be concerned about. Loss of more than two lines is the point that usually indicates a lack of success regarding the efficacy of these procedures [15–16].

### Statistical analysis

Statistical analysis was performed using SPSS statistical software, version 22.0 (IBM SPSS statistics). The t-test was used to compare ages between two categories of the dichotomous outcome dependent variables (safety and efficacy). Uni-variate analysis using Chi-Square test was performed to assess the effects on safety and efficacy of refractive surgeries by various qualitative variables (gender, treatment type, treatment period and SEQ before treatment). Multivariate analysis was performed using multiple logistic regression analysis, using the Forward Stepwise Likelihood Ratio method, to investigate the effects of several variables simultaneously, on the safety and efficacy indices. All P-values were 2 tailed and significance level of 0.05 or less was considered statistically significant.

## Results

We evaluated 8,775 eyes of 4,623 patients at the last follow-up visit, which took place 3–12 months after having refractive eye surgery. Of these, 2,197 were males (47.5%) and 2426 females (52.5%). The average age of the patients was 28.6 ± 8.7 years (range 17.3–67.2 years). The mean SEQ before treatment was -4.76 D (range [-16.02]-[-0.02] diopters). The mean central corneal thickness before treatment was 529.6 μ (range 442–654 μ). Off all eyes, 3,884 eyes had PRK surgeries (44.3%) while 4891 eyes had LASIK surgeries (55.7%) (Table 1).

**Table 1:** Characteristics of the study population.

Age, y (mean, SD)		28.6 ± 8.7
Gender		
	Female	4576 (52.1%)
	Male	4198 (47.8%)
SEQ, D (mean, SD)		-4.76 ± 2.38
Corneal thickness μ (mean, SD)	529.6 ± 33.8	
Procedure		
	PRK	3884 (44.3%)
	LASIK	4891 (55.7%)
Treatment period		
	2002–2006	4276 (48.7%)
	2007–2010	2883 (32.9%)
	2011–2015	1614 (18.4%)

SEQ = spherical equivalent; PRK = photorefractive keratectomy; LASIK = laser assisted in situ keratomileusis; SD = standard deviation

The pre-operative SEQ and treatment periods were divided into four categories. The pre-operative SEQ categories were 0 to (-3) D, (-3.01) to (-6) D, (-6.01) to (-9) D and higher than (-9.01) D (Table 2). Most eyes had SEQ up to (-6) (74.8%) and only a small group had SEQ higher than (-9.01) (6.1%). The surgeries were performed between 2002 and 2015 and treatment periods were divided into 3 time periods: 4276 eyes (48.7%) were operated during 2002–2006, 2883 eyes (32.95%) on 2007–2010 and 1614 eyes (18.4%) at 2011–2015 (Table 1).

**Table 2:** SEQ before treatment, categorized.

		Count	Percentage
SEQ	0 to (-3) D	2298	26.2%
	(-3.01) to (-6) D	4269	48.6%
	(-6.01) to (-9) D	1675	19.1%
	> -9 D	533	6.1%

SEQ = spherical equivalent

The major outcome measures were the safety index and efficacy index at last follow-up visit. The overall mean Safety Index for all procedures was 1.03±0.13 and the overall mean Efficacy Index for all procedures was 0.97±0.2. A cut-off level of 0.85 for the safety index and 0.80 for the efficacy index were set to determine successful results. Of all procedures, 715 (8.1%) were below the Safety cut-off level, and 1229 (14%) were below Efficacy cut-off level (Table 3).

**Table 3:** Success of safety and efficacy.

		Count	Percentage
Safety	Above cut-off (>0.85)	8060	91.9%
	Below cut-off (< = 0.85)	715	**8.1%**
Efficacy	Above cut-off (>0.8)	7546	86%
	Below cut-off (< = 0.8)	1229	**14%**

The effects of several dichotomous independent variables on the Safety and Efficacy indices were evaluated. First, univariate analysis was used to evaluate the effects of the different independent variables (such as age, gender, pre-operative SEQ, corneal thickness, treatment type and treatment period) on the Safety and Efficacy indices.

### Univariate analysis

#### Age at the time of surgery

Younger age was significantly correlated with safety and efficacy indices above the cut-off levels using a T-Test (P<0.001, 2-tailed). Mean age for patients above safety and efficacy cut-off levels (28.3±8.49, 28.0±8.20 years respectively) was significantly younger than for patients below cut-off levels (32.0±10.3, 32.4±10.58 years respectively) (Table 4).

**Table 4:** Correlation between age and safety and efficacy (T-Test).

		Age before treatment				
		N	Mean	Std. Deviation	Sig. (2-tailed)	
**Safety**	Below cut-off (< = 0.85)	714	32.02	10.303	**p<0.001**	
	Above cut-off (>0.85)	8039	28.31	8.49		
**Efficacy**	Below cut-off (< = 0.8)	1222	32.38	10.59	**p<0.001**	
	Above cut-off (>0.8)	7531	28.00	8.21	** **	

P-value indicates association between age and outcome above/below cut-off.

### Gender

Male gender was significantly correlated with efficacy above the cut-off level using Pearson Chi-Square test (P<0.001, 2-sided). 12.0% of male’s eyes were below efficacy cut-off level compared to 15.8% of female’s eyes (p<0.001). 7.9% of male’s eyes were below safety cut-off compared to 8.3% of female’s eyes (p = 0.5) (Table 5).

**Table 5:** Correlation between gender and safety and efficacy (Pearson Chi-Square).

			Safety			Efficacy		
			Below cut-off (< = 0.85)	Above cut-off (>0.85)	Sig. (2-sided)	Below cut-off (< = 0.8)	Above cut-off (>0.8)	Sig. (2-sided)
Gender	Male eyes	Percentage	333	3865	p = 0.5	503	3695	**p<0.001**
		Count	**7.90%**	92.10%		**12.00%**	88.00%	
	Female eyes	Percentage	381	4195		725	3851	
		Count	**8.30%**	91.70%		**15.80%**	84.20%	

P-value indicates association between gender and outcome above/below cut-off.

### SEQ before treatment

**Fig 1:**
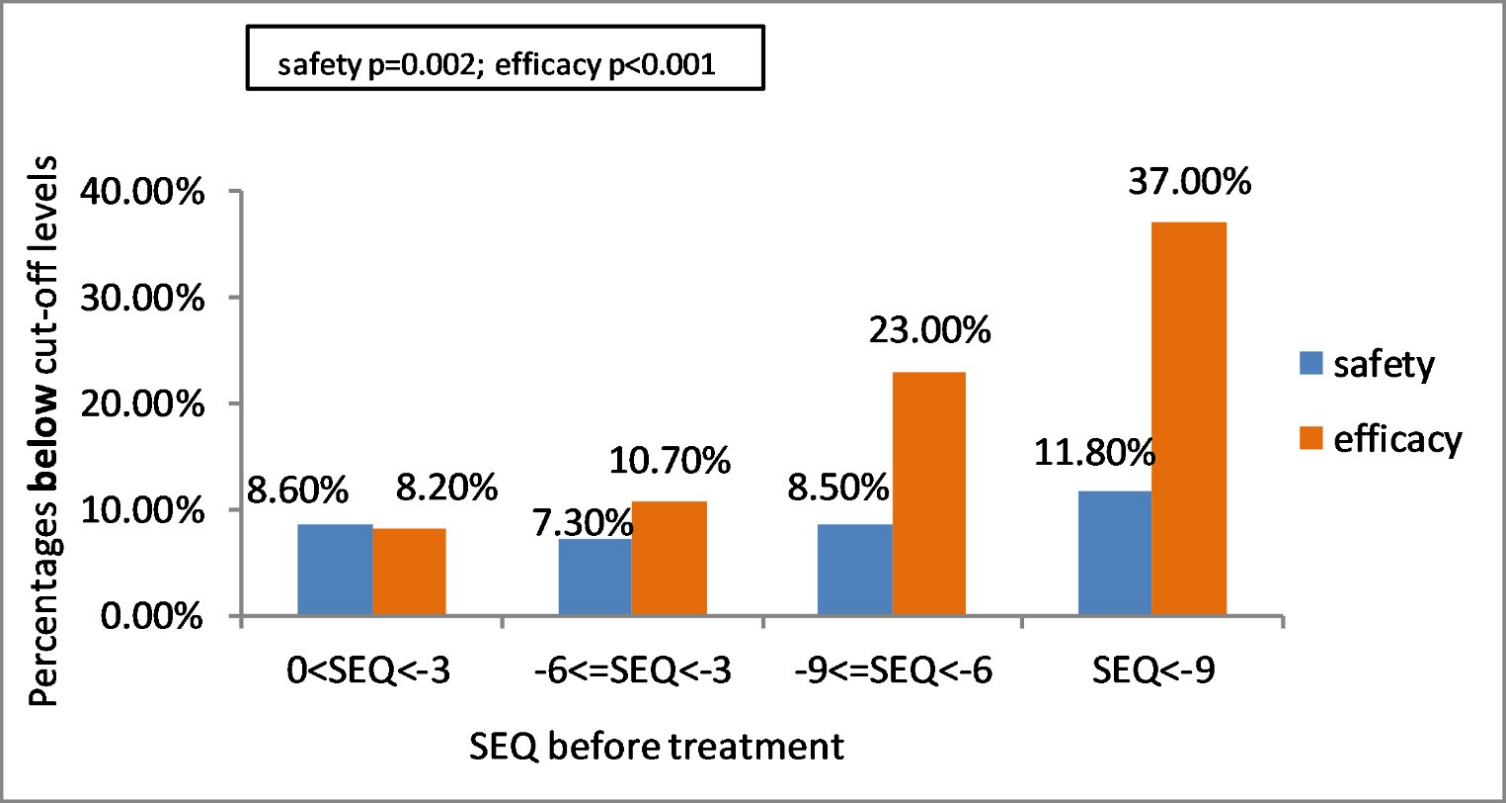
Association between SEQ before treatment and eyes below efficacy and safety cut-off levels using univariate analysis and Pearson Chi-Square test (p = 0.002 for safety, p<0.001 for efficacy). SEQ = spherical equivalent.

Refractive surgeries were performed on myopic eyes with SEQ ranging from 0 to -16 D. Lower SEQs were associated with both safety (p = 0.002) and efficacy (p<0.001) indices above the cut-offs using Pearson Chi-Square test. In the group of SEQ 0 to (-3) D 198 eyes (8.6%) were below the safety cut-off, in the group of SEQ (-3.01) to (-6) D 311 eyes (7.3%), in the group of SEQ (-6.01) to (-9) D 143 eyes (8.5%), and in the group of SEQ > -9 D 63 eyes (11.8%)–were below the safety cut-off (Table 6)(Fig 1).

**Table 6:** Correlation between SEQ before treatment and safety and efficacy (Pearson Chi-Square).

			Safety			Efficacy		
			Below cut-off (< = 0.85)	Above cut-off (>0.85)	Sig. (2-sided)	Below cut-off (< = 0.8)	Above cut-off (>0.8)	Sig. (2-sided)
SEQ before treatment	0 to (-3) D	Percentage	198	2100	**p = 0.002**	188	2110	**p<0.001**
		Count	**8.60%**	91.40%		**8.20%**	91.80%	
	(-3.01) to (-6) D	Percentage	311	3958		458	3811	
		Count	**7.30%**	92.70%		**10.70%**	89.30%	
	(-6.01) to (-9) D	Percentage	143	1532		386	1289	
		Count	**8.50%**	91.50%		**23.00%**	77.00%	
	SEQ >-9 D	Percentage	63	470		197	336	
		Count	**11.80%**	88.20%		**37.00%**	63.00%	

SEQ = spherical equivalent

P-value indicates association between SEQ before treatment and outcome above/below cut-off.

In the group of SEQ 0 to (-3) D 188 (8.2%) eyes were below efficacy cut-off, in the group of SEQ (-3.01) to (-6) D 458 eyes (10.7%), in the group of SEQ (-6.01) to (-9) D 386 eyes (23.0%) and 197 eyes (37.0%) in the group of SEQ> -9 D were all bellow the efficacy cut off level. Higher SEQ before treatment was significantly associated with lower efficacy (p<0.001) (Table 6) (Fig 1).

### Treatment type

**Fig 2:**
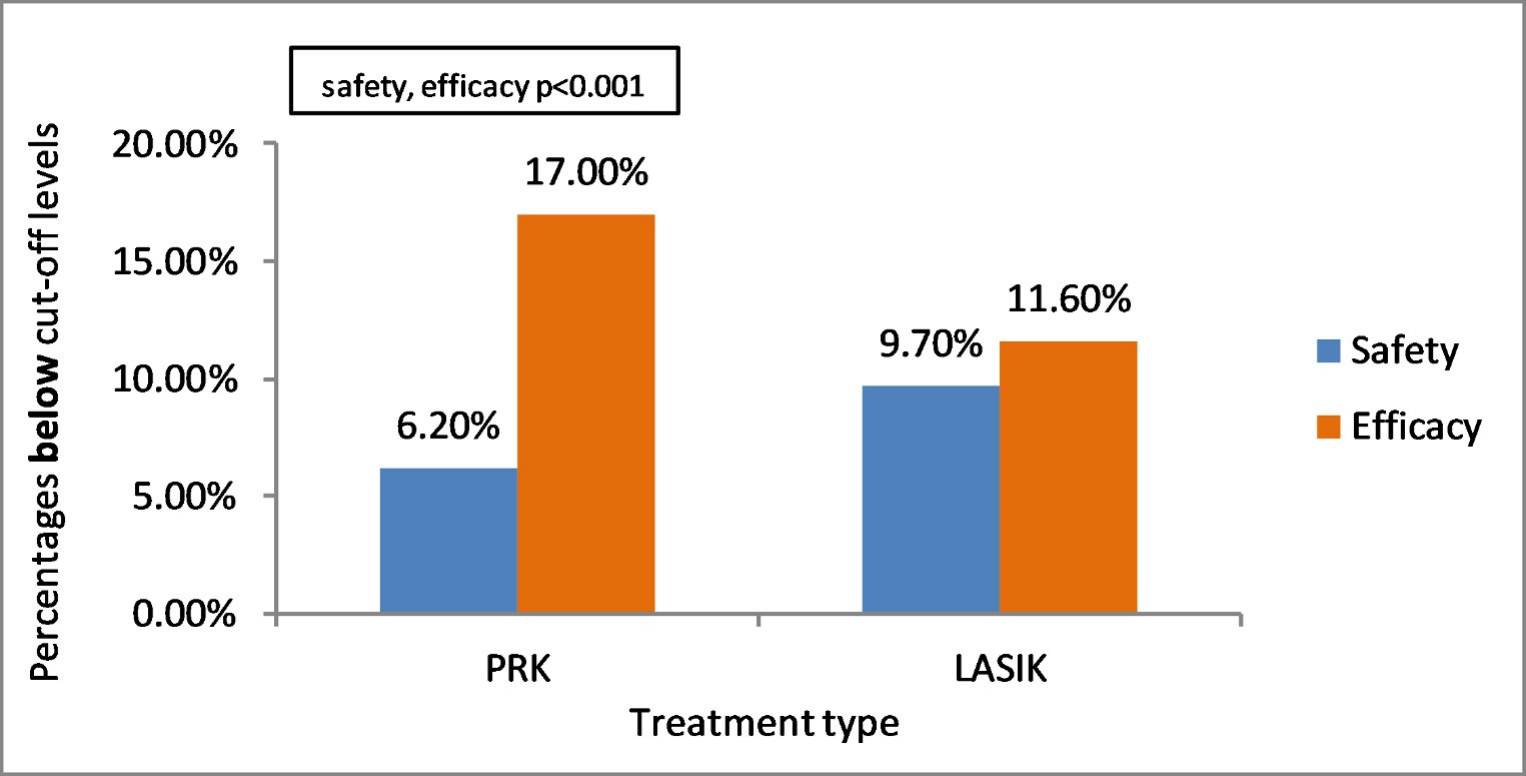
Association between treatment type and eyes below safety and efficacy cut-off levels using univariate analysis and Pearson Chi-Square test (p<0.001 for both safety and efficacy). PRK = photorefractive keratectomy; LASIK = laser assisted in situ keratomileusis.

Two types of refractive procedures were performed, PRK and LASIK. The surgical procedure was found to significantly affect the outcome: safety was found significantly higher in PRK, while efficacy was found significantly higher in LASIK using Pearson Chi-Square test (P<0.001, respectively). 6.2% of PRK surgeries were below safety cut-off level in comparison with 9.7% of LASIK surgeries (P<0.001). 17.0% of PRK surgeries were below efficacy cut-off levels in comparison with 11.6% of LASIK surgeries (P<0.001). (Table 7)(Fig 2).

**Table 7:** Correlation between treatment type and safety and efficacy (Pearson Chi-Square).

			Safety			Efficacy		
			Below cut-off (< = 0.85)	Above cut-off (>0.85)	Sig. (2-sided)	Below cut-off (< = 0.8)	Above cut-off (>0.8)	Sig. (2-sided)
Treatment type	PRK	Percentage	242	3642	**p<0.001**	660	3224	**p<0.001**
		Count	**6.2%**	93.8%		**17.00%**	83.00%	
	LASIK	Percentage	473	4418		569	4322	
		Count	**9.7%**	90.3%		**11.6%**	88.4%	

SEQ = spherical equivalent; LASIK = laser assisted in situ keratomileusis

P-value indicates association between treatment type and outcome above/below cut-off.

### Corneal thickness

Thinner corneas significantly correlated with the Safety index above the cut-off using T-test (p = 0.003, 2-tailed), but did not significantly affect Efficacy index (p = 0.263). Mean corneal thickness was 529μ for safety index above the cut-off level and 532μ below the cut-off level (Table 8).

**Table 8:** Correlation between corneal thickness and safety and efficacy (T-Test).

		Corneal thickness			
		N	Mean	Std. Deviation	Sig. (2-tailed)
**Safety**	Below cut-off (< = 0.85)	714	533.23	32.89	**p = 0.003**
	Above cut-off (>0.85)	8042	529.34	33.91	
**Efficacy**	Below cut-off (< = 0.8)	1229	528.65	34.56	**p = 0.263**
	Above cut-off (>0.8)	7527	529.82	33.73	** **

P-value indicates association between corneal thickness and outcome above/below cut-off.

### Treatment period

**Fig 3:**
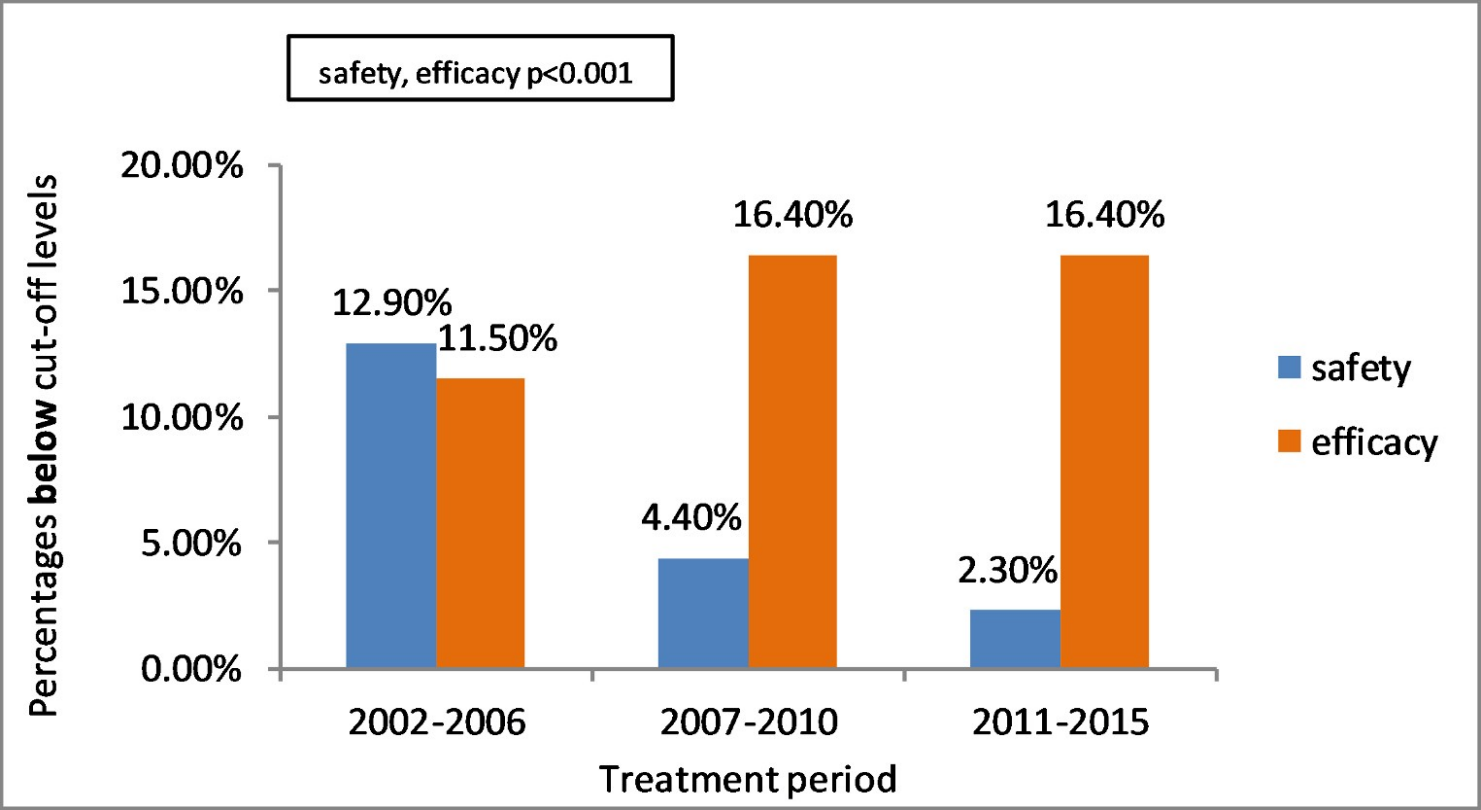
Association between treatment period and patients below safety and efficacy cut-off levels using univariate analysis and Pearson Chi-Square test (p<0.001 for both safety and efficacy).

Refractive surgeries were performed during 2002 to 2015. Safety index above the cut-off level was increased over the years while efficacy index was found higher in 2002–2006 than the later periods 2007–2010 and 2011–2015 using Pearson Chi-Square test (P<0.001, 2-sided). In 2002–2006 12.9% of safety indices were below cut-off level, 4.4% in 2007–2010 and 2.3% in 2011–2015. In 2002–2006 11.5% of efficacy indices were below cut-off level, 16.4% in 2007–2010 and 16.4% in 2011–2015 (Table 9)(Fig 3).

**Table 9:** Correlation between treatment period and safety and efficacy (Pearson Chi-Square).

			Safety			Efficacy		
			Below cut-off (< = 0.85)	Above cut-off (>0.85)	Sig. (2-sided)	Below cut-off (< = 0.8)	Above cut-off (>0.8)	Sig. (2-sided)
Treatment period	2002–2006	Percentage	551	3725	**p<0.001**	491	3785	**p<0.001**
		Count	12.9%	87.1%		11.5%	88.5%	
	2007–2010	Percentage	127	2756		472	2411	
		Count	4.4%	95.6%		16.4%	83.6%	
	2011–2015	Percentage	37	1577		264	1350	
		Count	2.3%	97.7%		16.4%	83.6%	

P-value indicates association between treatment period and outcome above/below cut-off.

### Multivariate analysis

Logistic Regression Model was used in order to evaluate the association between pre-operative factors (categorical and non-categorical) and the safety and efficacy indices. Only significant factors from the univariate analysis were tested in the logistic regression model.

### Safety

8,734 (99.5%) cases were analyzed out of the 8775 refractive procedures’ sample. 41 cases were excluded because of missing data, necessary for completing the multivariate analysis. Four significant factors were included in the Logistic Regression Model (Table 10):

**Table 10:** Logistic regression model–safety.

Variable		Sig.		Adjusted OR		95% C.I. for OR	
						Lower	Upper
**Age**			**p<0.001**		**0.969**	0.961	0.977
**SEQ before Treatment**	0 to (-3) D		**p<0.001**		**3.293**	2.367	4.581
	(-3.01) to (-6) D		**p<0.001**		**3.634**	2.651	4.981
	(-6.01) to (-9) D		**p<0.001**		**2.138**	1.533	2.982
	SEQ> -9 D		**p<0.001**		**1.0**	* reference category	
**Treatment period**	2002–2006		**p<0.001**		**1.0**	* reference category	
	2007–2010		**p<0.001**		**3.577**	2.889	4.429
	2011–2015		**p<0.001**		**7.598**	5.353	10.785

SEQ = spherical equivalent; OR = odds ratio; C.I = confidence interval

SEQ before treatment and treatment period—OR value is regarding the reference category. Age—OR value is relative to an increase of one year

#### Age

Increasing age was found associated to decreasing Safety with an OR (Odds Ratio) of 0.97 (95% CI 0.96–0.98) (p<0.001). OR value is relative to an increase of one year.

#### Treatment period

**Fig 4:**
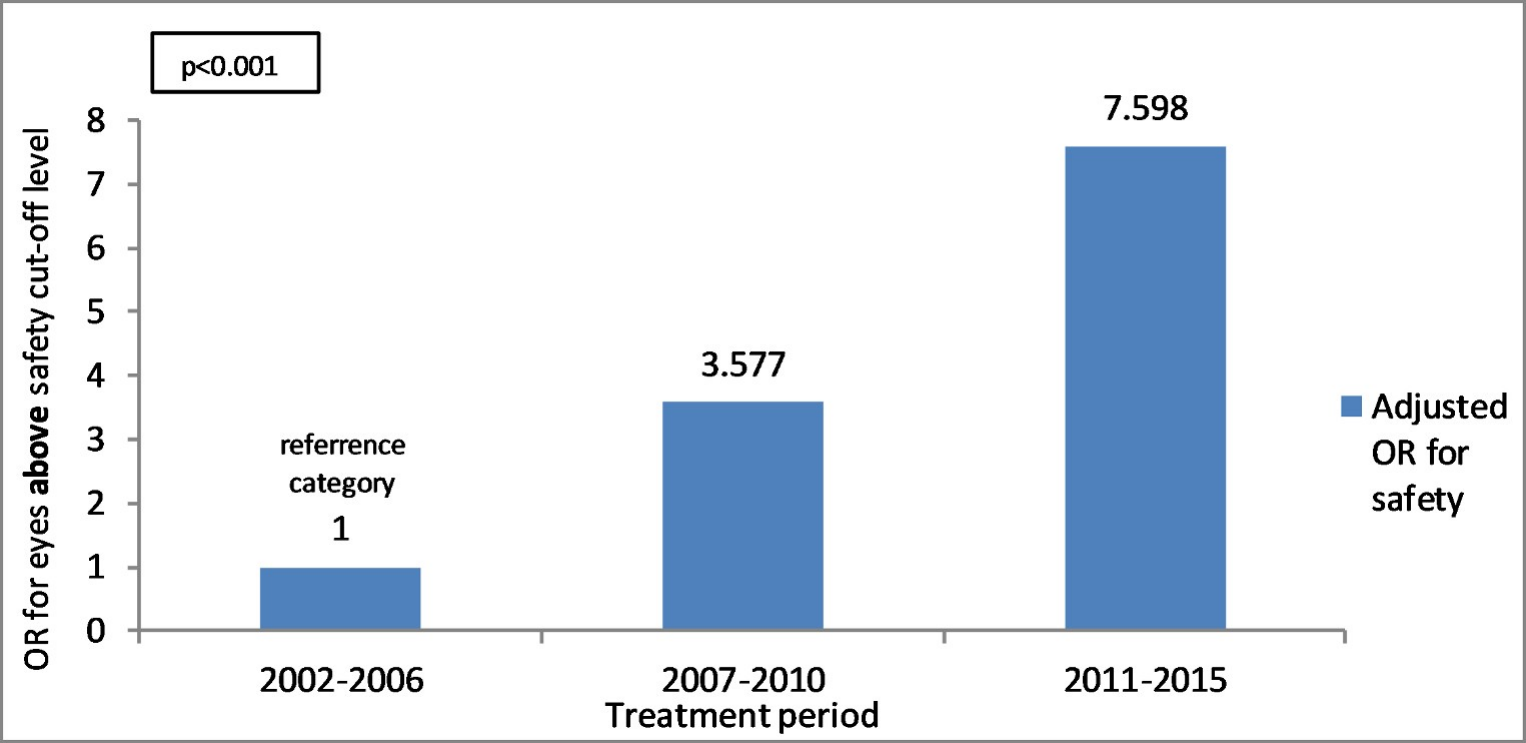
Relation between treatment periods and OR (Odds Ratio) for eyes above safety index cut-off level using multivariate analysis and logistic regression model (p<0.001). OR value is regarding the reference category.

Safety index above the cut-off level was found to be significantly increasing over the years. OR value is regarding the reference category of 2002–2006. The group of 2007–2010 has an OR of 3.58 (95% CI 2.89–4.43) (p<0.001) and the group of 2011–2015 has an OR of 7.59 (95% CI 5.35–10.78) (p<0.001) (Fig 4).

#### SEQ before treatment

**Fig 5:**
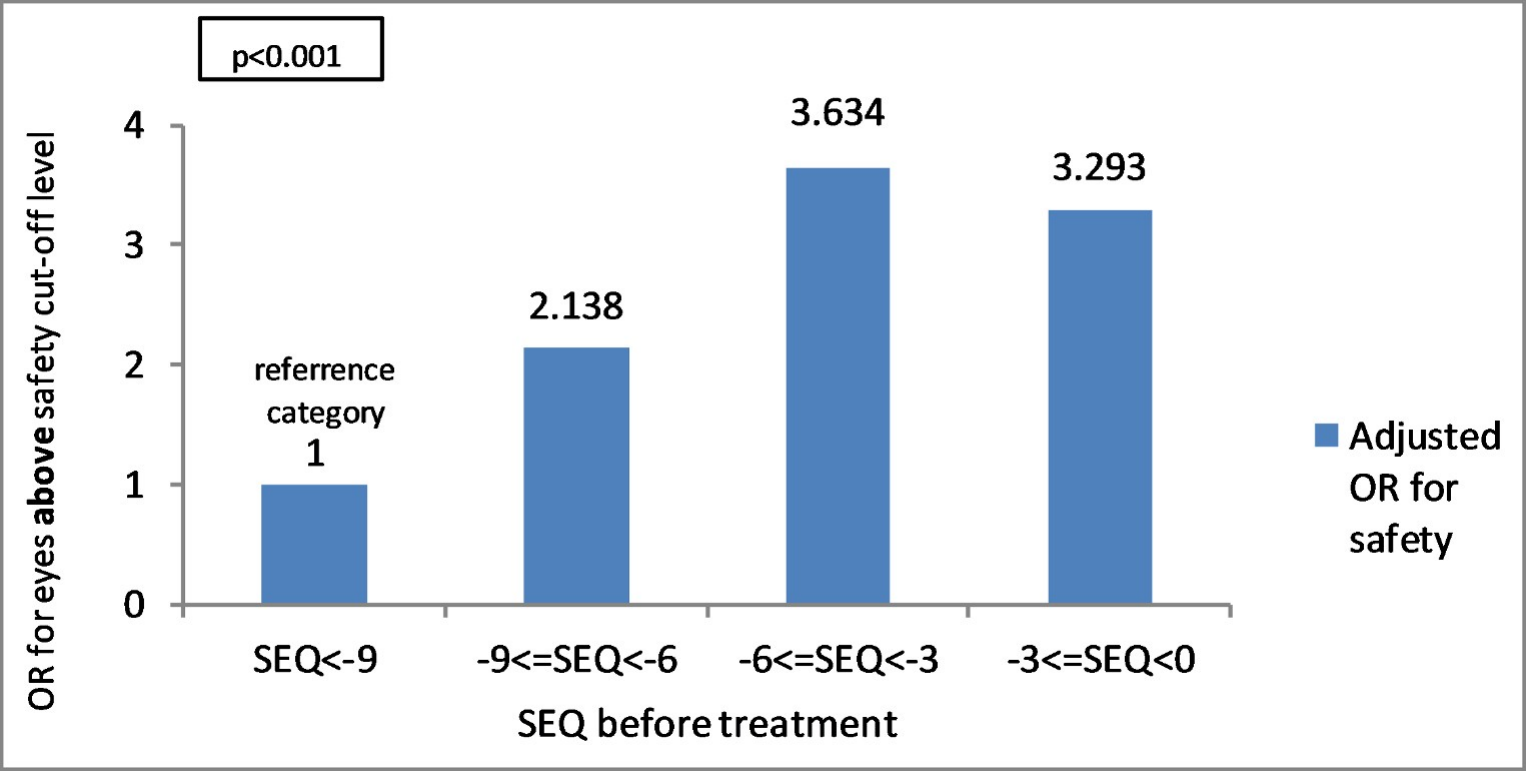
Relation between SEQ before treatment and OR for eyes above safety index cut-off level using multivariate analysis and logistic regression model (p<0.001). OR value is regarding the reference category. SEQ = spherical equivalent.

In myopic eyes, lower SEQ was found to be significantly associated with an increasing safety index above the cut-off level. OR value of 1 was assigned for the category of SEQ> -9 D. The group of SEQ between 0 to (-3) D had an OR of 3.29 (95% CI 2.37–4.58) (p<0.001), the group of SEQ (-3.01) to (-6) D had an OR of 3.63 (95% CI 2.65–4.98) (p<0.001) and the group of SEQ (-6.01) to (-9) D had an OR of 2.14 (95% CI 1.53–2.98) (p<0.001) (Fig 5).

#### Corneal thickness

Was not associated with a safer outcome (p = 0.48).

### Efficacy

8,752 (99.1%) cases were analyzed out of the 8775 refractive procedures’ sample. 23 cases were excluded because of missing data, necessary for completing the multivariate analysis. Four significant factors were included in the Logistic Regression Model (Table 11): age, treatment period, pre-operative SEQ and gender.

**Table 11:** Logistic regression model–efficacy.

		Sig.	Adjusted OR	95% C.I. for OR	
Variable				Lower	Upper
**Age**		**p<0.001**	0.943	0.937	0.949
**SEQ before Treatment**	0 to (-3) D	**p<0.001**	6.420	5.018	8.214
	SEQ >-3 D to -6 D	**p<0.001**	4.823	3.891	5.979
	SEQ >-6 D to -9 D	**p<0.001**	1.895	1.523	2.356
	SEQ> -9 D	**p<0.001**	1.0	* reference category	
**Treatment period**	2002–2006	**p<0.001**	1.0	* reference category	
	2007–2010	**p<0.001**	0.711	0.611	0.828
	2011–2015	**p = 0.017**	0.804	0.673	0.962
**Gender**	Male	**p = 0.017**	1.170	1.028	1.332

SEQ = spherical equivalent; OR = odds ratio; C.I = confidence interval

SEQ before treatment and treatment period—OR value is regarding the reference category. Age—OR value is relative to an increase of one year. Gender–OR value is regarding female gender.

#### Age

Increasing age was found associated to decreasing efficacy with an OR of 0.94 (95% CI 0.937–0.95) (p<0.001). OR value is relative to an increase of one year.

#### Treatment period

**Fig 6:**
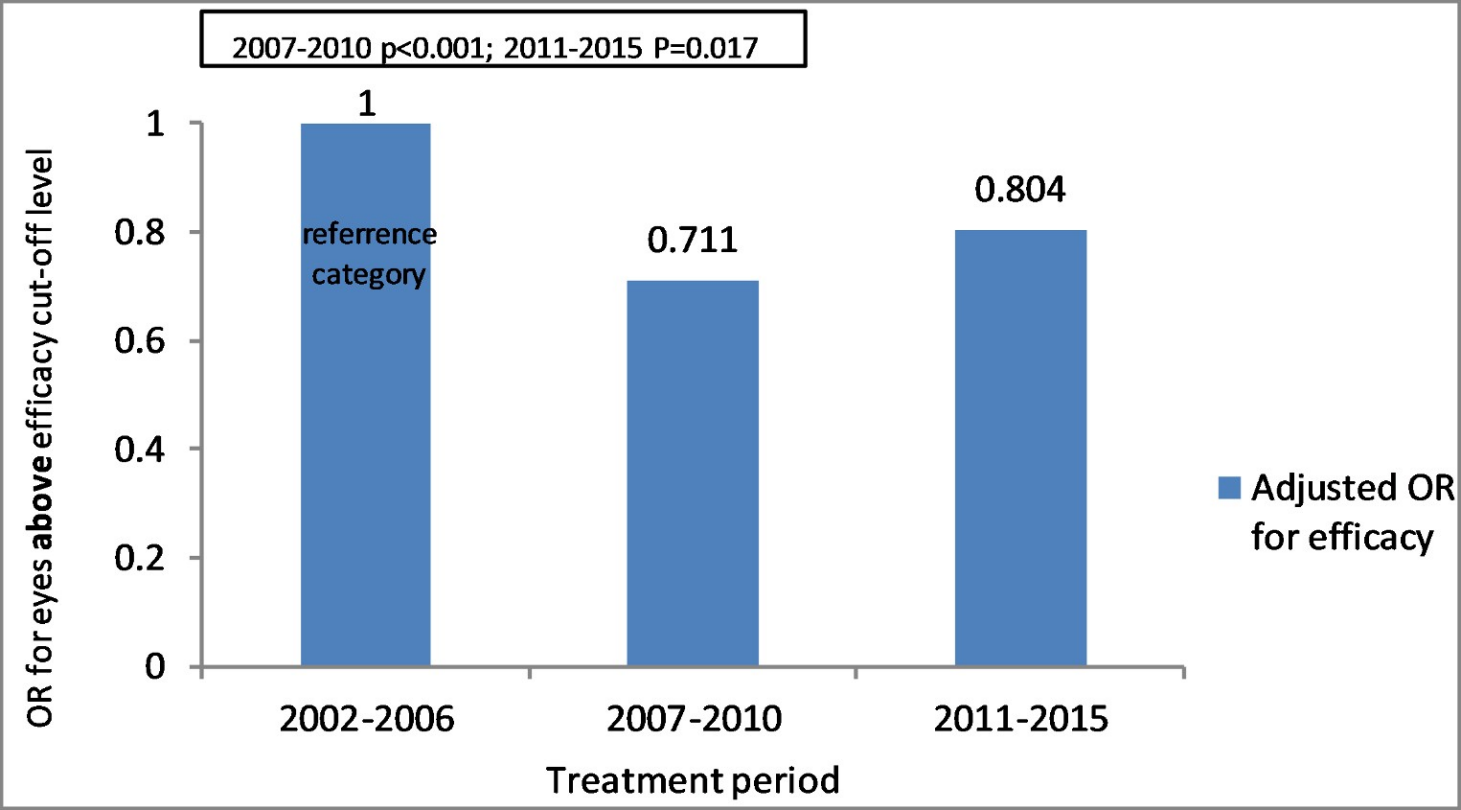
Relation between treatment period and OR for eyes above efficacy index cut-off level using Multivariate analysis and Logistic regression model (p<0.001 for 2007–2010 period; P = 0.017 for 2011–2015 period). OR value is regarding the reference category.

Efficacy index above cut-off level was found higher in 2002–2006 than the later periods 2007–2010 and 2011–2015. OR value is regarding the reference category of 2002–2006. The group of 2007–2010 has an OR of 0.71 (95% CI 0.61–0.83) (p<0.001) and the group of 2011–2015 has an OR of 0.804 (95% CI 0.673–0.962) (p = 0.017) (Fig 6).

#### SEQ before treatment

**Fig 7:**
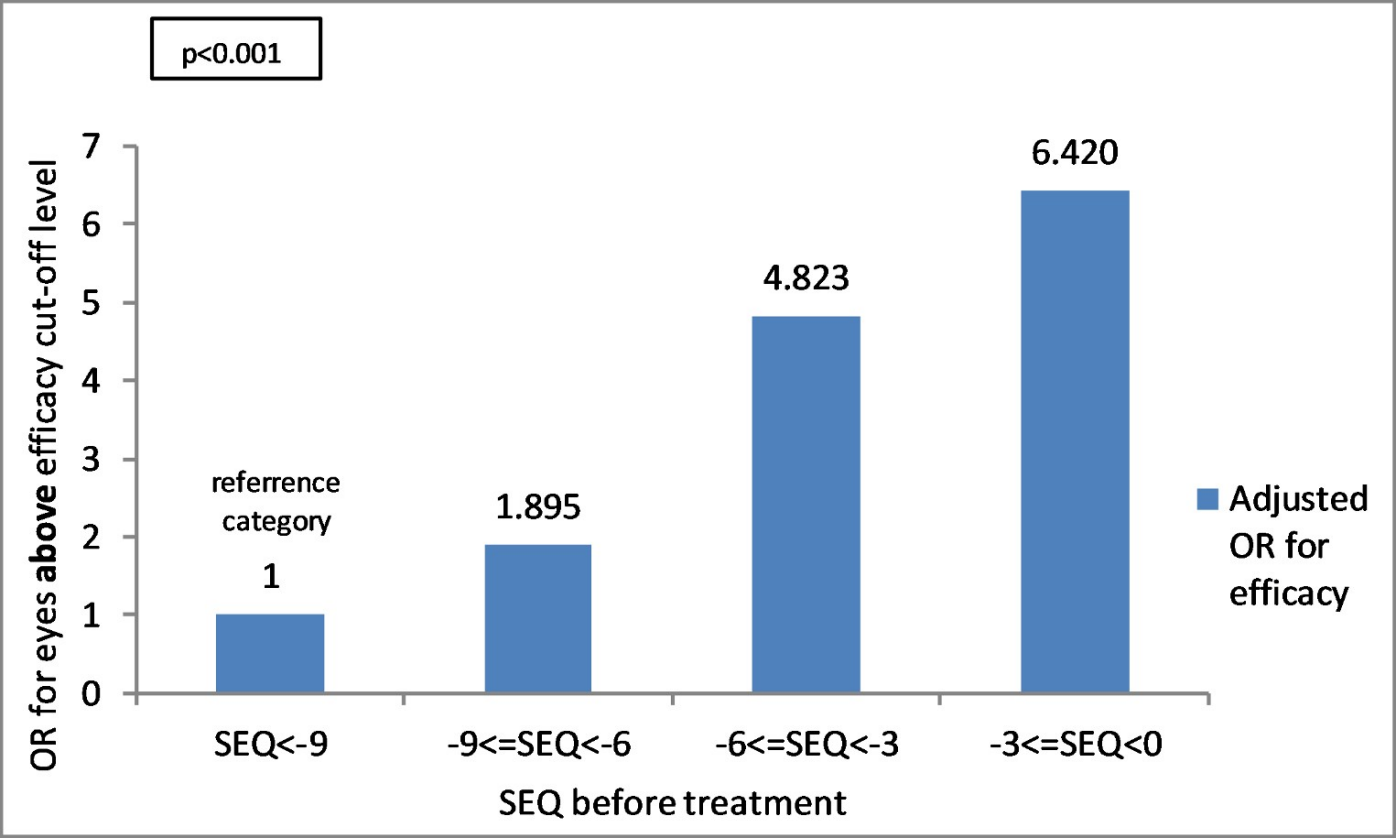
Relation between SEQ before treatment and OR for eyes above efficacy index cut-off level using multivariate analysis and logistic regression model (p<0.001). OR value is regarding the reference category. SEQ = spherical equivalent.

In myopic eyes, low SEQ was found to be associated with a higher efficacy index above cut-off level. OR value is regarding the reference category of SEQ > -9 D. The group of SEQ between 0 to (-3) D has an OR of 6.42 (95% CI 5.02–8.21) (p<0.001), the group of SEQ (-3.01) to (-6) D has an OR of 4.82 (95% CI 3.89–5.98) (p<0.001) and the group of SEQ (-6.01) to (-9) D has an OR of 1.89 (95% CI 1.52–2.36) (p<0.001) (Fig 7).

#### Gender

The Male gender was found weakly associated to increasing efficacy index above cut-off level with an OR of 1.17 (95% CI 1.03–1.33) (p = 0.017). OR value is regarding the reference category of female.

#### Treatment type

Multiple regression analysis found no differences of safety and efficacy between LASIK and PRK (P = 0.201).

## Discussion

Refractive surgery techniques have become a common treatment for the correction of refractive errors in myopic eyes. Outcomes, differences between PRK and LASIK procedures, and post-operative complications and their treatments are commonly reported. However, no study investigating the various predictive factors for success in safety and efficacy in these procedures has been published so far. Therefore, in our study, we evaluated the predicting factors by using a novel outcome method of preset cut-off levels (0.85 and 0.8 respectively). The strength of this study was by using a relatively large prospective sample size, including data available for 8,775 refractive procedures, performed in the same institute during the span of 13 consecutive years by a small fixed group of surgeons.

Complications of refractive surgeries were not discussed in this paper. We believe that complications should be investigated and addressed separately, and merit separate studies, devoted just to the understanding of those complications. Retreatment procedures, although performed by our team, were excluded from this study as it was not our main goal and should be discussed separately. As described before, our novel concept was to evaluate the overall predictive factors for efficacy and safety instead of discussing different complications and long term outcomes. Our goal in this study was rather to focus on factors that affect the overall result in a large cohort of patients. According to our belief, the success in safety and efficacy of these surgeries resulted from the different pre-operative factors that are investigated in our study rather than major complications.

An accurate knowledge of the safety and efficacy of refractive surgery procedures is a basic requirement for each refractive surgery service. Each refractive surgeon must be able to analyze the results of his procedures for the purpose of improving his practice, as well as for the need for patient information prior to deciding to undergo a refractive procedure. In our study we used the safety and efficacy indices for these purposes. These parameters give a simple, accurate and inclusive information on the safety and efficacy of refractive procedures, without specifically analyzing individual complications and side effects which might occur in various occasions. We believe that complications and side effects of refractive surgery may affect these indices, and may be reflected in the overall outcome.

In this study, analyzing 8,775 refractive surgeries for myopia, efficacy was found to be significantly correlated with younger age, male gender and low myopia. Safety was found to be significantly correlated with younger age, low myopia and to be significantly increasing over the years. Corneal thickness was found to be correlated with safety only by using uni-variate analysis. PRK procedures were safer compared to LASIK, while LASIK procedures were more effective compared to PRK procedures, using the uni-variate analysis. However, multiple regression analysis found no differences of safety and efficacy between these two refractive procedures. 91.9% and 86.0% of all evaluated eyes were above the safety and efficacy cut-off levels, respectively.

This study is the first to evaluate predictive factors for success in safety and efficacy of refractive surgeries using a novel evaluation system, for the analysis of a prospective large study cohort. In our study, the influence of confounders such as surgeons and equipment was minimized by having a small and fixed team of surgeons performing surgeries using similar techniques over the years. A new cut-off method was used for evaluating predictive factors, indicating percentages below and above cut-off levels of the safety and efficacy indices. Describing surgeries outcomes using cut-off levels is very meaningful to clinicians. Cut-off levels of 0.85 for the Safety index and 0.80 for the Efficacy index were set. Values below the safety 0.85 cut-off level indicates loss of two or more lines that is not acceptable for the BSCVA after an elective refractive surgery. Values below the efficacy 0.8 cut-off level indicates loss of more than two lines of UCVA, a level which may be unsatisfactory for patients anxious to achieve an optimal level of UCVA.

### Age

Younger age was significantly correlated with safety and efficacy indices above the cut-off levels using uni and multivariate analysis. This result can be attributed to healing properties of the cornea which are influenced by epithelial migration and proliferation processes, which are known to be better and faster in younger patients [17–20]. In younger people there is a higher amount of tears, the corneal sensitivity is higher and supports epithelial cells viability and proliferation. It was found that healing is slower and less extensive in mature corneas than in younger ones [17], older individuals have substantially slower recovery rates [18] and the aged cornea is slower to recover from hypoxic stress [20]. Currently there is no accepted upper age limit for refractive surgeries, and in our study only 13 patients (0.14%) were above sixty years old, none of whom had suffered from cataract or nuclear sclerosis. Younger patients under the age of eighteen were operated before military service only if their spherical equivalent was stable for at least twelve months.

### Pre-operative SEQ

In this study myopic eyes with lower SEQ were associated with both safety and efficacy indices above the cut-off levels. It is generally accepted that eyes with mild myopia have better BCVA and UCVA outcomes after having refractive surgeries than eyes with high myopia. Similar results were previously reported [4,21–23]–a better UCVA at 6–12 months post-treatment was found in low to moderate myopia rather than moderate to high myopia [4] and higher percent with UCVA of 20/20 or better was found in low myopic eyes [22].

### Refractive procedure

We found no differences between these two refractive procedures regarding safety and efficacy. Using the multiple regression analysis, the safety and efficacy of these procedures were similar, although in the uni-variate analysis PRK was found safer, while LASIK was found more effective. There is controversy concerning safety and efficacy of those two procedures. Many studies claim LASIK is safer and more efficient over PRK while others claim they are comparable in all aspects [4,24]. The differences may be caused by the change in different evaluation techniques. A comparison between the two techniques found similarity in safety and efficacy, however found that PRK induced statistically fewer higher-order aberrations than LASIK at 6 months [25]. The multiple regression analysis used in this study found no significant difference between the two procedures in similarity with most recent studies [4,9,26]. The different results in this study may be caused using uni-variate and multiple regression analysis.

### Treatment period

Safety index above the cut-off level was significantly increased over the years. This is most probably explained by the increasing experience of the surgeons over the years, by performing better preoperative screening of patients with known risk factors for ectasia such as keratoconus, familial keratoconus history, and avoiding surgery on thin corneas and high myopia [4]. The use of Wavefront technology and newer laser technologies had contributed to the improved safety over the years as also reported in previous studies [4,22]. Increased safety over the years could be also explained by using newer models of microkeratomes: specifically replacing the Hansatome microkeratome (Bausch & Lomb, Rochester, NY) used in LASIK surgeries with the newer Bausch & Lomb XP Microkeratome. Efficacy was found higher in the early period of 2002–2006 in comparison to later periods. This may reflect the increased proportion of PRK procedures compared to LASIK procedures at our institute during the years. In addition, the low OR value in this analysis indicates a weak correlation, as reflected by the relatively high P-value of 0.017 (although significant, this level of significance was generally much lower compared to the significance levels for the other parameters). In our study, the targeted refraction (the target refraction after surgery) remained unchanged during the entire study period. Younger patients were targeted to a slight overcorrection of +0.25 D while adults above 40 years were targeted to 0 D.

### Gender

Male gender was found significantly correlated with efficacy above the cut-off level. This is the first study demonstrating such significant result by using a prospective large study cohort. This difference however, was found to have a much lower significance level on the multivariate analysis (p = 0.017), suggesting that it may be a coincident finding, resulting from the likelihood of finding significance by chance when using a large number of variables in a large cohort study. We could not find a reasonable explanation for these data, and could not find another study supporting this observation.

### Corneal thickness

In this study corneal thickness did not correlate with efficacy index above cut-off level and thicker corneas were found to have a negligible effect on safety index above cut-off level. This may reflect a tendency of our surgeons to prefer performing PRK surgery in patients with thinner corneas or to avoid operating patients with very thin corneas.

In summary, our large prospective study found that Efficacy in refractive surgery for myopia is significantly correlated with younger age, male gender and low myopia. Safety was correlated with younger age, low myopia and increases over the years with surgical experience. Multiple regression analysis found no differences of safety and efficacy between PRK and LASIK procedures. Corneal thickness did not affect the efficacy of refractive procedures and had a negligible effect on the safety in our study.

Our study has some limitations—We did not include data on complications and retreatments, as our aim was to focus mainly on the predictive factors, while avoiding the conventional outcome presentation charts. In addition, our study included a relatively long period in which important technological changes occurred in this field. However, we used this potential limitation to look at the effect of the surgery time period as one of the predictive variables. Moreover, we had a relatively high percentage of excluded patients due to missing data, as this is the nature of retrospective studies. A small portion of the study cohort had follow-up time of less than six months and even smaller portion with less than 3 months, however we expected only a slight difference between the results of six and twelve months [14].

This is the first study to use a large refractive surgery cohort of patients, evaluating pre-operative predictive factors for safety and efficacy in laser refractive surgeries for myopia, using a novel cut-off method for evaluating these factors. We believe that our results will help clinicians in better understanding the effects of the different pre-operative parameters on the success of these procedures, and will enable better pre-operative consultation, and improved decision making and patients selection. Further studies are needed to improve our understanding of the predictive factors for success in refractive surgeries for longer follow up periods, using our new method to predict safety and efficacy.

## Supporting information

S1 Dataset.xlsx This file contains the data of the patients. (XLSX)

## Abbreviations

PRK: PhotoRefractive Keratectomy
LASIK: Laser ASsisted In situ Keratomileusis
SEQ: Spherical EQuivalent
OR: Odds Ratio

## References

[1] Sandoval HP, de Castro LEF, Vroman DT, Solomon KD. Refractive surgery survey 2004. Journal of Cataract & Refractive Surgery 2005; 31: 221–233. 15721716 10.1016/j.jcrs.2004.08.047

[2] Mitchell P, Hourihan F, Sandbach J, Wang JJ. The relationship between glaucoma and myopia: The Blue Mountains Eye Study. Ophthalmology 1999; 106: 2010–2015. 10519600 10.1016/s0161-6420(99)90416-5

[3] Xu L, Wang Y, Wang S, Wang Y, Jonas JB. High myopia and glaucoma susceptibility: the Beijing Eye Study. Ophthalmology 2007; 114: 216–220. 10.1016/j.ophtha.2006.06.050 17123613 10.1016/j.ophtha.2006.06.050

[4] Shortt AJ, Allan BDS, Evans JR. Laser‐assisted in‐situ keratomileusis (LASIK) versus photorefractive keratectomy (PRK) for myopia. The Cochrane Library 2013. 10.1002/14651858.CD005135.pub3 PMC11848121 23440799

[5] Sakimoto T, Rosenblatt MI, Azar DT. Laser eye surgery for refractive errors. The Lancet 2006; 367: 1432–1447. 10.1016/S0140-6736(06)68275-5 16650653

[6] Ehlke GL, Krueger RR. Laser Vision Correction in Treating Myopia. The Asia-Pacific Journal of Ophthalmology 2016;5: 434–437. 10.1097/APO.0000000000000237 27898448 10.1097/APO.0000000000000237

[7] Hammond MD, Madigan WP, Bower KS. Refractive surgery in the United States Army, 2000–2003. Ophthalmology 2005;112: 184–190. 10.1016/j.ophtha.2004.08.014 15691549 10.1016/j.ophtha.2004.08.014

[8] Ortega-Usobiaga J, Llovet-Osuna F, Djodeyre MR, Llovet-Rausell A, Beltran J, Baviera J. Incidence of corneal infections after laser in situ keratomileusis and surface ablation when moxifloxacin and tobramycin are used as postoperative treatment. Journal of Cataract & Refractive Surgery 2015; 41.6: 1210–1216. 26096523 10.1016/j.jcrs.2014.09.041

[9] Kuryan J, Cheema A, Chuck RS. Laser-assisted subepithelial keratectomy (LASEK) versus laser‐assisted in‐situ keratomileusis (LASIK) for correcting myopia. The Cochrane Library 2017. 10.1002/14651858.CD011080.pub2 PMC5408355 28197998

[10] Linke SJ, Baviera J, Munzer G, Fricke OH, Richard G, Katz T. Mesopic pupil size in a refractive surgery population (13,959 eyes). Optometry and Vision Science 2012; 89.8: 1156–1164. 22773178 10.1097/OPX.0b013e318263c165

[11] de Rojas V, Llovet F, Martínez M, Cobo-Soriano R, Ortega-Usobiaga J, Beltran J, et al Infectious keratitis in 18 651 laser surface ablation procedures. Journal of Cataract & Refractive Surgery 2011; 37.10: 1822–1831. 21865006 10.1016/j.jcrs.2011.04.037

[12] Albelda-Vallés JC, Martin-Reyes C, Ramos F, Beltran J, Llovet F, Baviera J. Effect of preoperative keratometric power on intraoperative complications in LASIK in 34,099 eyes. Journal of Refractive Surgery 2007; 23.6: 592–597. 17598579 10.3928/1081-597X-20070601-10

[13] Saw SM, Gazzard G, Shih‐Yen EC, Chua WH. Myopia and associated pathological complications. Ophthalmic and Physiological Optics 2005; 25: 381–391. 10.1111/j.1475-1313.2005.00298.x 16101943 10.1111/j.1475-1313.2005.00298.x

[14] Shahinian L. Laser-assisted subepithelial keratectomy for low to high myopia and astigmatism. Journal of Cataract & Refractive Surgery 2002; 28.8: 1334–1342. 12160801 10.1016/s0886-3350(02)01444-x

[15] Al-Tobaigy FM. Efficacy, predictability, and safety of laser-assisted subepithelial keratectomy for the treatment of myopia and myopic astigmatism. Middle East African journal of ophthalmology 2012; 19.3: 304. 22837624 10.4103/0974-9233.97931 PMC3401800

[16] Reinstein DZ, Archer TJ, Randleman JB. JRS standard for reporting astigmatism outcomes of refractive surgery. Journal of Refractive Surgery 2014; 30.10: 654–659. 25291747 10.3928/1081597X-20140903-01

[17] Staatz WD, Van Horn DL. The effects of aging and inflammation on corneal endothelial wound healing in rabbits. Investigative ophthalmology & visual science 1980; 19: 983–986. 7409992

[18] Polse KA, Brand R, Mandell R, Vastine D, Demartini, Flom R. Age differences in corneal hydration control. Investigative ophthalmology & visual science 1989; 30: 392–399. 2925313

[19] Lass JH, Greiner JV. The effects of age on phosphatic metabolites of the human cornea. Cornea 1995;14:89–94. 7712743

[20] Faragher RGA, Mulholland B, Tuft SJ, Sandeman S, Khaw PT. Aging and the cornea. British journal of ophthalmology 1997; 81: 814–817. 9486017 10.1136/bjo.81.10.814 PMC1722015

[21] Yuen LH, Chan WK, Koh J, Mehta JS, Tan DT. A 10-Year Prospective Audit of LASIK Outcomes for Myopia in 37932 Eyes at a Single Institution in Asia. Ophthalmology 2010; 117: 1236–1244. 10.1016/j.ophtha.2009.10.042 20153899 10.1016/j.ophtha.2009.10.042

[22] Bailey MD, Zadnik K. Outcomes of LASIK for myopia with FDA-approved lasers. Cornea 2007; 26: 246–254. 10.1097/ICO.0b013e318033dbf0 17413947 10.1097/ICO.0b013e318033dbf0

[23] Dutt S, Steinert RF, Raizman MB, Puliafito CA. One-year results of excimer laser photorefractive keratectomy for low to moderate myopia. Archives of Ophthalmology 1994; 112: 1427–1436. 7980132 10.1001/archopht.1994.01090230041018

[24] Shortt AJ, Bunce C, Allan BDS. Evidence for superior efficacy and safety of LASIK over photorefractive keratectomy for correction of myopia. Ophthalmology 2006; 113: 1897–1908. 10.1016/j.ophtha.2006.08.013 17074559 10.1016/j.ophtha.2006.08.013

[25] Moshirfar M, Schliesser JA, Chang JC, Oberg TJ, Mifflin MD, Townley R, et al Visual outcomes after wavefront-guided photorefractive keratectomy and wavefront-guided laser in situ keratomileusis: prospective comparison. Journal of Cataract & Refractive Surgery 2010; 36: 1336–1343. 20656157 10.1016/j.jcrs.2010.02.012

[26] Yang KJ, Yan HT, Nakahori Y. Evaluation of the effectiveness of laser in situ keratomileusis and photorefractive keratectomy for myopia: a meta-analysis. Journal Of Medical Investigation 2003; 50: 180–186. 13678388

